# Antimicrobial Resistance in *Streptococcus pneumoniae* before and after the Introduction of Pneumococcal Conjugate Vaccines in Brazil: A Systematic Review

**DOI:** 10.3390/antibiotics13010066

**Published:** 2024-01-09

**Authors:** Patricia Alice Knupp-Pereira, Amanda Seabra Cabral, Ítalo Moraes Dolores, Amanda Beiral da Silva, Helvécio Cardoso Correa Póvoa, Felipe Piedade Gonçalves Neves

**Affiliations:** 1 Instituto Biomédico, Universidade Federal Fluminense, Niterói 24020-150, Brazil; patricia_alice@id.uff.br (P.A.K.-P.); amandaseabra@id.uff.br (A.S.C.); amandabeiral@id.uff.br (A.B.d.S.); 2 Faculdade de Medicina, Universidade de São Paulo, São Paulo 01246-903, Brazil; italodolores@usp.br; 3 Instituto de Saúde de Nova Friburgo, Universidade Federal Fluminense, Nova Friburgo 28625-650, Brazil; hpovoa@id.uff.br

**Keywords:** *Streptococcus pneumoniae*, antimicrobial resistance, pneumococcal conjugate vaccine, invasive pneumococcal disease, pneumococcal colonization

## Abstract

*Streptococcus pneumoniae* causes serious illnesses, such as pneumonia, bacteremia, and meningitis, mainly in immunocompromised individuals and those of extreme ages. Currently, pneumococcal conjugate vaccines (PCVs) are the best allies against pneumococcal diseases. In Brazil, the 10-valent and 13-valent PCVs have been available since 2010, but the threat of antimicrobial resistance persists and has been changing over time. We conducted a systematic review of the literature with works published since 2000, generating a parallel between susceptibility data on isolates recovered from colonization and invasive diseases before and after the implementation of PCVs for routine childhood use in Brazil. This systematic review was based on the Cochrane Handbook for Systematic Reviews of Interventions and Preferred Reporting Items for Systematic Literature Reviews and Meta-Analyses (PRISMA) guidelines. Despite the inclusion of PCVs at a large scale in the national territory, high frequencies of non-susceptibility to important drugs used in pneumococcal diseases are still observed, especially penicillin, as well as increasing resistance to macrolides. However, there are still drugs for which pneumococci have a comprehensive sensitivity profile.

## 1. Introduction

*Streptococcus pneumoniae* is a common colonizer of the human upper respiratory tract. However, pneumococci can cause milder diseases, such as acute otitis media (AOM) and sinusitis, as well as severe diseases, including community-acquired pneumonia (CAP), bacteremia, and meningitis, affecting individuals of all age groups, especially those of extremes ages and immunocompromised people [1,2,3,4].

The main prevention strategy against pneumococcal diseases is pneumococcal conjugate vaccines (PCVs). They confer a high degree of protection against specific serotypes, interfering in the fluctuation of their distribution and in the prevalence of resistance to antimicrobial agents [5,6]. There are several PCVs approved for use in children and adults in different countries [7,8,9,10,11,12,13]. In Brazil, the 7-valent PCV (PCV7; serotypes 4, 6B, 9V, 14,18C, 19F, and 23F) was initially made available, in 2001, in private immunization clinics for children and in the Brazilian public health system (*Sistema Único de Saúde*, SUS) for children < 5 years old who were at high risk of invasive pneumococcal diseases (IPD). In 2010, the 10-valent PCV (PCV10; PCV7 serotypes + 1, 5, and 7F) was introduced into the Brazilian National Immunization Program (NIP) for free-of-charge immunization of all children < 5 years old. Initially, the PCV10 schedule comprised three primary doses at 2, 4, and 6 months of age and a booster dose at 12–15 months of age (3p + 1), but, since 2016, the PCV10 dosing regimen in the Brazilian NIP changed to 2p + 1 (at 2 and 4 months of age and a booster dose at 12 months of age). In 2010, the 13-valent PCV (PCV13; PCV10 serotypes + 3, 6A, and 19A) replaced PCV7 in private clinics, and it was made available via SUS in 2019 for individuals aged 5 years or older who are at the highest risk for IPD, including patients living with HIV/AIDS, patients with cancer, and those who underwent solid organ or bone marrow transplantations. In 2023, the PCV15 (PCV13 serotypes + 22F and 33F) was approved for use in Brazil [13,14,15,16,17,18,19].

Although not compulsory, vaccination in Brazil is strongly recommended. The most used antipneumococcal vaccine in Brazil is PCV10, and between 2011 and 2022, considering the different geographical regions, the average PCV10 vaccination coverage with primary doses and the booster dose was 88.5% and 80.6%, respectively. Data on PCV13 coverage are limited, but a few studies report a low coverage (<8%) among children < 5 years old [20,21,22].

Some post-PCV10 introduction studies in Brazil indicate a reduction in the average mortality rate of pneumonia (11%; from 29.69 to 23.40 per 100,000) in children younger than 1 year after four years of vaccination [23] and a significant reduction, between 13.9% and 17.6%, in hospitalizations for pneumonia in the target groups of vaccination over five years after PCV10 implementation [24]. On the other hand, the incidence of pneumococcal meningitis remains high in Brazil, with approximately 1000 cases/year [25].

Beta-lactams, especially penicillin and amoxicillin, are the main, but not exclusive, choices to treat pneumococcal diseases. Other antimicrobial agents frequently used against pneumococcal diseases include macrolides, fluoroquinolones, and lincosamides [26,27,28]. The first choice for AOM is amoxicillin, which may be combined with clavulanate in cases of recurrence within 30 days or when associated with other symptoms. For allergic people, cefdinir or azithromycin has been frequently prescribed [29]. For CAP patients without comorbidities, the most indicated treatment includes amoxicillin, doxycycline, or a macrolide, and for those with comorbidity, a fluoroquinolone or a combination of amoxicillin with clavulanate or cephalosporin plus a macrolide or doxycycline. Furthermore, for patients admitted to a hospital, a fluoroquinolone in monotherapy or the combination of a macrolide with a beta-lactam is recommended, with a difference in treatment for patients in intensive care, who require the combination of a beta-lactam with a macrolide or a fluoroquinolone [30].

Antimicrobial resistance, however, is a concern among *S. pneumoniae*. Penicillin non-susceptible pneumococci (PNSP) are considered a medium priority risk to human health by the World Health Organization [31]. Drug-resistant *S. pneumoniae* is also classified as a serious threat in the USA [32]. The growing report of resistance to different antimicrobial agents has been a cause for concern in public health and demands strategies in public policies, as well as therapeutic alternatives [27].

This systematic literature review aims to verify the Brazilian scenario pre- and post-PCV10 regarding antimicrobial resistance among *S. pneumoniae* isolates associated with colonization and diseases recovered from individuals from all age groups. For this reason, we selected the year 2000 as a starting point, considering that it corresponds to 10 years before the introduction of PCV10 in Brazil.

## 2. Results

**Figure 1:**
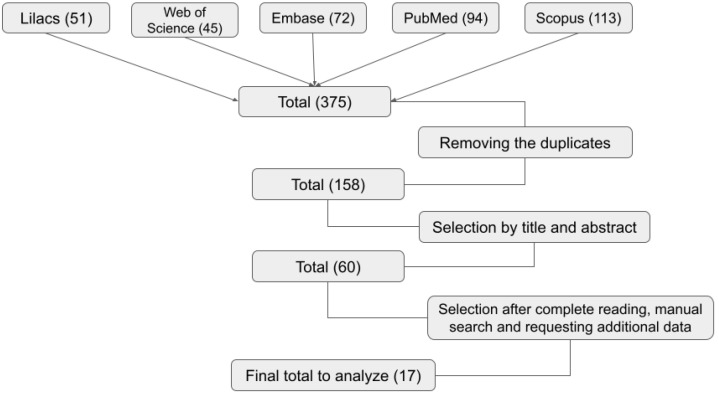
Detailed flowchart for obtaining and selecting eligible articles for this systematic review.

We obtained 375 references between articles and academic works through searches in the databases mentioned in Section 4. Among these, according to the inclusion criteria, seventeen references [21,22,33,34,35,36,37,38,39,40,41,42,43,44,45,46,47] were selected to retrieve data according to the scheme in Figure 1. Files referring to data from the SIREVA II Program of the Pan American Health Organization are available in WHO electronic domains [48,49,50,51,52,53,54,55,56,57,58,59,60,61,62].

### 2.1. Assessment of the Methodological Quality of the Articles

Regarding the description and case definition of the population of the studies, only two (11.8%) of seventeen articles were negatively classified. Seven (41.2%) of the seventeen articles described the representativeness of the sample and its sampling in a clear way. All articles described the type of test used and mentioned or referenced the evaluative standard used. However, only five (29.4%) articles described the use of internal quality control. Detailed data can be found in Table 1.

### 2.2. Data Extraction

Of the 17 articles selected for this systematic review [21,22,33,34,35,36,37,38,39,40,41,42,43,44,45,46,47], the main information extracted from them is presented in Table 2, divided into parts: a (pre-PCV10 period), b (post-PCV10 period), and c (extended period; those with data from both pre- and post-PCV10 periods).

Considering all the references included in this study, we obtained data on 18,273 isolates; data on 15,437 (84.5%) isolates were provided by SIREVA II (invasive isolates) and data on 2839 (15.5%) isolates were obtained through the included articles. Of 18,273 isolates, 2683 (14.7%) isolates were associated with colonization, 117 (0.6%) isolates with non-invasive diseases, and 39 (0.2%) isolates were associated with invasive diseases, but not presented by SIREVA II. In total, 8991 (49.2%) isolates were from the pre-PCV10 period and 9285 (50.8%) were from the post-PCV10 period.

Invasive isolates included those from sterile sites, such as blood, pleural fluid, and cerebrospinal fluid (CSF). The colonization isolates, obtained through the articles, were mainly collected through sterile swabs in contact with the nasopharynx and oropharynx. Other types of isolates were included in non-invasive pneumococcal diseases, such as ear abscess, cervical abscess, buttock abscess, nasal/eye abscess, bronchial aspirate, corneal aspirate, sinus aspirate, pulmonary aspirate, tracheal aspirate, sputum, bronchoalveolar lavage, auricular secretion, bronchial secretion, tear duct secretion, conjunctival secretion, ocular secretion, wound secretion, skin secretion, pulmonary secretion, postauricular secretion, tracheal secretion, rectal swab, corneal ulcer, and urine.

Antimicrobial resistance data were compiled and organized into tables separated by pre- and post-PCV10 introduction periods (Table 3 and Table 4).

Higher frequencies of resistance to sulfamethoxazole-trimethoprim were observed in invasive isolates in the pre-PCV10 period (60.1%; 4815/8016). No case of non-susceptibility (intermediate + resistant) in the pre-PCV10 period was observed for vancomycin, linezolid, trovafloxacin, telithromycin, and quinupristin-dalfopristin, as well as resistance to amoxicillin. In the post-PCV10 introduction period, no resistance was observed to vancomycin, linezolid, telithromycin, and quinupristin-dalfopristin.

Data on susceptibility to penicillin and ceftriaxone were separated into meningitis and non-meningitis and by period, respectively, in Table 5(a,b) and Table 6(a,b).

For ceftriaxone, we observed a higher proportion of resistance to general in the post-PCV10 introduction period (6.5%, 25/384), similar to penicillin, which showed a higher proportion (44.6%; 499/1118).

Regarding macrolide resistance, a greater volume of data were obtained for erythromycin. There is a high susceptibility for colonization isolates in the pre-PCV10 period (95.2%; 719/755), with a decline in susceptibility in the post-PCV10 period (82%; 596/727). These findings were similar to invasive isolates, which in the pre-PCV10 period were 94.5% susceptible (6795/7186) and 81.1% (6397/7884) in the post-PCV10 period.

Finally, a small number of isolates was tested against fluoroquinolones and, as a result, there are data on ofloxacin and trovafloxacin susceptibility only for the pre-PCV10 period. All ninety-two (100%) carriage isolates tested against ofloxacin and the two (100%) carriage isolates tested against trovafloxacin were susceptible. For invasive (*n* = 1) and non-invasive (*n* = 2) disease isolates, the susceptibility against trovafloxacin was also 100%. Levofloxacin had a higher number of susceptible isolates, with a proportion of 98.8% (557/564) in the pre-PCV10 period and 100% (565/565) in the post-PCV10 period for colonization isolates. All the 20 invasive isolates from the pre-PCV10 period were susceptible to levofloxacin. All non-invasive disease isolates from the pre-PCV10 (48/48) and post-PCV10 (22/22) periods were also susceptible to levofloxacin.

### 2.3. Statistical Analysis

**Figure 2:**
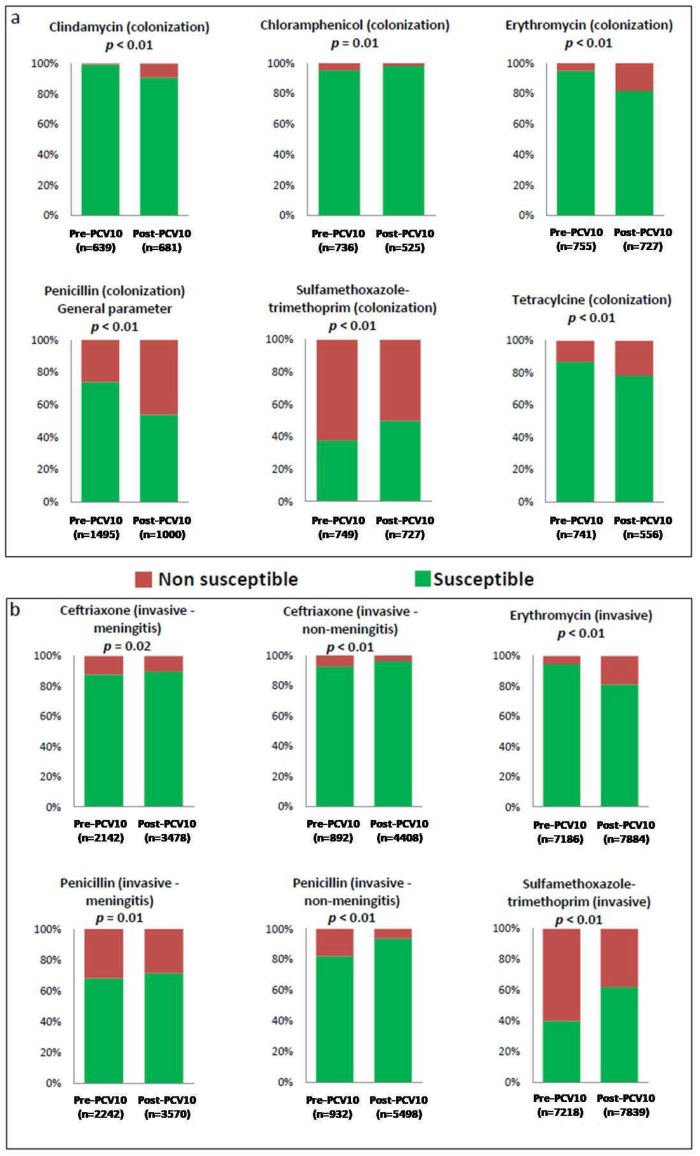
The proportion of isolates susceptible and non-susceptible to antimicrobial agents according to isolation source ((**a**). carriage isolates; (**b**). invasive isolates) before and after the introduction of the 10-valent pneumococcal conjugate vaccine (PCV10) for universal use in Brazil (*p*-value was calculated using a two-proportion *Z*-test to compare independent samplings).

The proportion of erythromycin non-susceptible isolates was higher among carriage (*p* < 0.01) and invasive (*p* < 0.01) isolates of the post-PCV10 period. The proportion of sulfamethoxazole-trimethoprim susceptibility (*p* < 0.01) was higher among isolates of the post-PCV10 period, regardless the isolation source. Although a limited number of isolates has been tested against meropenem, susceptibility to this drug was higher among non-invasive isolates (*p* < 0.01) of the post-PCV10 period. Among carriage isolates, the frequencies of susceptibility to chloramphenicol (*p* = 0.01), as well as non-susceptibility to clindamycin (*p* < 0.01) and tetracycline (*p* < 0.01), were higher after PCV10 introduction. Among invasive isolates (meningitis and non-meningitis), the proportion of susceptibility to penicillin (*p* ≤ 0.01) and ceftriaxone (*p* ≤ 0.02) was higher after PCV10 introduction. On the other hand, the frequency of penicillin non-susceptible pneumococci was higher among carriage isolates (*p* < 0.01) in the general parameter in the post-PCV10 period. Figure 2 shows the main results of proportion tests when statistically significant differences in the antimicrobial susceptibility profile were detected between isolates of the pre- and post-PCV10 periods.

## 3. Discussion

Based on the 17 articles selected through this systematic literature review, a high number of articles (88.2%; 15/17) were positively classified within the tool used (modified Newcastle–Ottawa assessment scale) [63,64,65], offering greater reliability in the use of the data obtained. Notably, a considerably high number of invasive isolates originated from SIREVA II (84.4%, 15,437/18,276), considered an important epidemiological surveillance tool for *S. pneumoniae* and other microorganisms in Latin America.

The susceptibility to sulfamethoxazole-trimethoprim (SXT) was higher after PCV10 introduction for routine use in Brazil (*p* = 0.01). In the pre-PCV10 period among invasive isolates, the proportion of SXT susceptibility and non-susceptibility was 40.3% (2907/7218) and 59.7% (4311/7218), respectively. In the post-PCV10 introduction period, there was a drop in the number of non-susceptible isolates (37.7%; 2957/7839) compared to the susceptible ones (62.3%; 4882/7839). This comparison is interesting because it presents a change in the general panorama of antimicrobial resistance of this drug, tending to a drop in resistance levels. However, it is noteworthy that this phenomenon is not uniformly observed in other countries; for example, a recent study carried out in Malawi (southeast Africa) with colonization and invasive isolates verified a high frequency of resistance to SXT (96%; 137/143), with similar resistance profiles worldwide [66].

For penicillin, there was a statistically significant difference in the percentage of non-susceptibility between the pre- and post-PCV10 introduction periods among invasive isolates, with lower frequencies for both meningitis (31.9% to 28.7%; *p* = 0.01) and non-meningitis (17.7% to 6.3%; *p* < 0.01) after PCV10 use. This finding is very important since in Latin America most countries usually report a prevalence of penicillin resistance among meningitis isolates over 30% [62]. On the other hand, regarding the general parameter, there was an increase in non-susceptibility between the same periods from 25.9% (387/1495) to 44.1% (461/1000) for colonization isolates (*p* < 0.01), respectively. This may be explained mainly by the impact of childhood vaccination with PCV10 in Brazil since before PCV10 introduction, resistance to beta-lactams was mostly associated with serotypes included in the vaccine formulation, especially 6B, 14, 19F, and 23F [38,40,67]. After PCV10 introduction, these serotypes were nearly eliminated from both colonization and diseases [21,22,67].

Due to the serotype replacement phenomenon, some of the main serotypes circulating in Brazil are currently 19A in invasive diseases with high resistance to different classes of antimicrobial agents and 6C in colonization isolates [21,36,40,44]. In this context, a replacement by PCV13, PCV15, PCV20, and even Pneumosil^®^, which also protects against 10 vaccine serotypes, would be appropriate to replace PCV10 in the Brazilian National Immunization Program [6,9,10,11,19]. However, this phenomenon may continue due to the varied range of capsular serotypes and their distribution among populations.

For ceftriaxone, the general parameter shows a higher frequency of non-susceptible isolates (4.6%; 25/542) in isolates associated with colonization in the post-PCV10 introduction period. Although not statistically significant (*p* = 0.43), this is of paramount importance since third-generation cephalosporins are frequently used to treat pneumococcal meningitis [26], and isolates with this resistance profile circulating within a population represent a high risk of transmission and development of severe diseases. In turn, susceptibility to ceftriaxone was significantly higher (*p* ≤ 0.02) among invasive isolates recovered in the post-PCV10 period.

Frequencies of susceptibility to macrolides, namely erythromycin and clarithromycin, exceeded 70% across all periods evaluated. A similar profile between invasive and colonization isolates was observed, with an important decline in susceptibility in the post-PCV10 period. Resistance in the pre-PCV10 period was around 5% for both colonization and invasive isolates. However, the proportion of macrolide-resistant isolates almost reached 20% in the post-PCV10 period. Macrolide resistance has been increasing worldwide. A nationwide surveillance in the USA between 2018 and 2019, with isolates recovered from blood and respiratory specimens from adults, revealed a high burden of macrolide resistance among *S. pneumoniae*, reaching almost 40% [68].

Levofloxacin is the fluoroquinolone with the greatest amount of data available for analysis, and the authors observed a high proportion of susceptibility among colonization isolates in the pre-PCV10 (98.8%; 557/564) and the post-PCV10 (100%; 565/565) periods. A similar scenario was observed for invasive isolates, in which all isolates (24 isolates from pre-PCV10 and 50 isolates from post-PCV10 periods) were susceptible to fluoroquinolones.

Despite the increasing and worrying resistance to beta-lactams and macrolides, all isolates were susceptible to vancomycin, linezolid, telithromycin, and quinupristin-dalfopristin in both the pre- and post-PCV10 introduction periods.

The main limitation of this work was the high variation of data presentation in the articles included in this review, making it difficult to group them. Also, 22 articles with important data were not made available in time by the authors, despite attempts to contact them. Still, we retrieved data on an extensive collection of isolates recovered from various clinical sources, mainly associated with IPD, and from different geographical regions of Brazil, providing a comprehensive scenario of antimicrobial resistance in pneumococci before and after PCV introduction for routine use in Brazil.

## 4. Materials and Methods

### 4.1. Search Strategy

This systematic review was structured between May 2022 and July 2023 with the search date on 23 May 2023. The following databases were consulted: Lilacs (Latin American & Caribbean Health Sciences Literature), Embase, Pubmed, Scopus, and Web of Science. In addition to these, a manual search was carried out in the bibliographic references of the selected articles and data extraction from the documents was produced by the System of Surveillance Networks of Responsible Agents for Bacterial Pneumonia and Meningitis (SIREVA II; Electronic page: https://www3.paho.org/hq/index.php?option=com_docman&view=list&slug=sireva-ii-8059&Itemid=270&lang=pt#gsc.tab=0; accessed on 23 May 2023).

The files referring to the search strategies according to the base can be found in the Supplementary Material as Table S1 and the manual search as Table S2.

This review was based on the question: “How is the resistance profile to antimicrobial agents of Streptococcus pneumoniae isolates before and after the introduction of pneumococcal conjugate vaccines in Brazil?”. It is noteworthy that this research was submitted to the Prospero platform [PROSPERO acknowledgment of receipt (364743)].

### 4.2. Article Selection and Data Extraction

All articles found were initially evaluated based on titles and abstracts. After this step, some articles were selected for full reading based on the inclusion and exclusion criteria listed in Table 7.

Two authors performed these steps and a third author was consulted in case of doubt. Then, data were extracted using the Microsoft Excel^®^ program.

### 4.3. Quality Assessment

Individual quality control of each academic work was evaluated according to the Newcastle–Ottawa Quality Tool Assessment Scale with modifications according to models by Sugianli et al., 2021 and Mancini et al., 2017 for cross-sectional studies and according to data demand [63,64,65].

### 4.4. Data Compilation

The data obtained were compiled and analyzed using Excel^®^, allowing the division of data according to the vaccination period: pre- or post-PCV introduction in the Brazilian immunization program.

In the case of penicillin and ceftriaxone (beta-lactams), from 2007 onwards, the evaluation parameters were divided into two groups: meningitis and non-meningitis [69]. Data from articles with these definitions were added to their respective classifications (meningitis and non-meningitis). Articles that did not use the parameters listed above for beta-lactams were assigned to the general parameter column for better organization and analysis of the data. Furthermore, when the data were provided by the authors (raw data), in the case of penicillin specifically, originating from sources of colonization (non-invasive), the parameters of oral penicillin were used and the data were added in the general column; when invasive, meningitis and non-meningitis parameters were used. In the case of ceftriaxone, meningitis criteria were applied for invasive isolates and non-meningitis for colonization isolates.

Articles presenting data from a long time covering both periods (pre- and post-PCV10 introduction) had their data organized separately.

We retrieved data for the following classes of antimicrobial agents: amphenicols (chloramphenicol), macrolides (erythromycin, clarithromycin), sulfonamides and diaminopyrimidines (sulfamethoxazole-trimethoprim), glycopeptides (vancomycin), lincosamides (clindamycin), tetracyclines (tetracycline), ansamycins (rifampicin), fluoroquinolones (levofloxacin, ofloxacin, gemifloxacin, trovafloxacin), oxazolidinones (linezolid), ketolides (telithromycin), streptogramins (quinupristin-dalfopristin), and beta-lactams (penicillin, amoxicillin, meropenem, cefotaxime, cefuroxime, oxacillin).

The data obtained were divided into a table with three different parts, namely, part a (pre-PCV10 period), referring to academic works prior to 2010 [34,35,36,37,39,40,43,47]; b (post-PCV10 period), composed of articles with data after the same year [21,22,38,41,42,44,45,46]; and c (extended period), formed by works that contain data from both periods [33].

It is also noteworthy that among the works that required data supplementation, we received only the raw data from the article by Pinto et al. 2019 [33] on time for this study. In this context, the data were separated into three distinct groups according to the scope of this systematic literature review.

### 4.5. Statistical Analysis

We used a two-proportion *Z*-test to compare independent samplings, at a confidence level of 95%, and verify if the proportion of pneumococci non-susceptible to antimicrobial agents has significantly changed in the post-PCV10 period.

### 4.6. Ethical Aspects

All included studies were approved by their respective Ethics Committees. Other data were retrieved from a public database.

## 5. Conclusions

There is evidence that the proportion of isolates that are susceptible to chloramphenicol and sulfamethoxazole-trimethoprim is higher after PCV10 implementation for routine use in Brazil. More importantly, the same scenario was observed for penicillin and ceftriaxone among isolates associated with IPD. However, it is important to highlight the higher frequency of penicillin non-susceptible pneumococci associated with colonization in the post-PCV10 introduction period due to the emphasis on its use in the treatment of pneumococcal diseases. The emergence of macrolide-resistant isolates, associated with both colonization and diseases, is also a concern. Similarly, resistance to clindamycin and tetracycline is significantly higher among carriage isolates of the post-PCV10 period. On the other hand, susceptibility to other antimicrobial agents, such as ansamycins, fluoroquinolones, glycopeptides, and oxazolidinones, remains high, making them available as alternatives for use as monotherapy or in combined therapy.

## Figures and Tables

**Table 1:** Methodological quality assessment through the modified Newcastle–Ottawa assessment scale [63] adapted from Sugianli et al., 2021 [64] and Mancini et al., 2017 [65].

Newcastle—Ottawa	Selection		Comparability	Outcome			Total	Classification
Article	1	2	1	1	2	3	Number of “Yes”/Total	
Pinto et al., 2019 [38]	No	No	No	Yes	Yes	No	2/6	Poor
Velasquez et al., 2009 [39]	Yes	Yes	Yes	Yes	Yes	Yes	6/6	Excellent
Pereira et al., 2004 [40]	Yes	Yes	Yes	Yes	Yes	Yes	6/6	Excellent
Cardozo et al., 2006 [41]	Yes	Yes	No	Yes	Yes	No	4/6	Good
Franco et al., 2010 [42]	Yes	Yes	Yes	Yes	Yes	No	5/6	Excellent
Neves et al., 2019 [43]	Yes	Yes	No	Yes	Yes	No	4/6	Good
Neves et al., 2017 [21]	Yes	Yes	No	Yes	Yes	No	4/6	Good
Reis et al., 2008 [44]	Yes	Yes	Yes	Yes	Yes	No	5/6	Excellent
Neves et al., 2013 [45]	Yes	Yes	No	Yes	Yes	No	4/6	Good
Laranjeira, 2014 [46]	Yes	Yes	No	Yes	Yes	Yes	5/6	Excellent
Da Silva et al., 2023 [47]	Yes	Yes	Yes	Yes	Yes	No	5/6	Excellent
Fortuna et al., 2023 [22]	Yes	Yes	No	Yes	Yes	No	4/6	Good
Fonseca et al., 2005 [48]	Yes	Yes	No	Yes	Yes	No	4/6	Good
Brandileone et al., 2019 [49]	Yes	Yes	Yes	Yes	Yes	No	5/6	Excellent
Zanella et al., 2019 [50]	Yes	Yes	Yes	Yes	Yes	No	5/6	Excellent
Rezende et al., 2021 [51]	Yes	Yes	No	Yes	Yes	No	4/6	Good
Laval et al., 2006 [52]	Yes	Yes	No	Yes	Yes	No	4/6	Good

Selection (definition of population): (1) Is the study population clearly described? (2) Are the criteria for enrollment in the study clearly defined? Comparability (sample representativeness): (1) Is the sample of the target population clearly described? Outcome (Antimicrobial susceptibility testing method verification): (1) Does the study describe the type of susceptibility testing used? (2) Did the study specify the test standard used? (3) Did the study describe an internal quality control measure?

**Table 2:** (a) Main results retrieved from articles with data of the pre-PCV10 period. (b) Main results retrieved from articles with data of the post-PCV10 period. (c) Main results retrieved from article with data of the extended period (covering pre- and post-PCV10 period) with raw data provision.

Reference	Collection Date	City of Study	Clinical Source	Age Range	Study Scenario	Number of Isolates and Main Findings
(a)						
[34]	April to October 2008	Umuarama	NP swab Colonization	Children aged 3 months to 6 years	Nine daycare centers.	92 isolates from 570 children Full susceptibility to LEV, LIN, ofloxacin, RIF, telithromycin, and VAN. Non-susceptibility frequencies: - CHL (1.1%), CLI (1.1%), ERY (8.7%), MDR (9.8%), PEN-I (34.8%), PEN-R (22.8%), SXT (72.8%), and TET (6.5%).
[35]	June 2001 to October 2002	Porto Alegre	Children’s middle ear effusion (NIPD)	Children aged 11 months to 10 years	Pediatric otorhinolaryngology outpatient clinic.	*S. pneumoniae* was detected by PCR in 16 (12.5%) and by culture in 8 (6.3%) of 128 clinical specimens. Of 8 isolates: 3 (37.5%) PEN-S, 3 (37.5%) PEN-I, and 2 (25%) PEN-R.
[36]	November 2002 to July 2003	Salvador	NP swab Colonization	Adolescents: 37.3% (aged 10–13 years), 49.4% (aged 14–16 years), and 13.3% (aged 17–19 years)	Public schools.	83 isolates Full susceptibility to CHL, CLI, RIF, and VAN. No resistance to CTX in 18 isolates tested. Non-susceptibility frequencies: - ERY (4.8%), PEN-I (7.2%), SXT (37.3%), and TET (18.1%).
[37]	August to December 2005	Goiânia	NP swab Colonization	Children aged 2 to 59 months	62 of the 70 municipal daycare Centers.	686 isolates Full susceptibility to LEV and VAN. - PNSP: 178 (25.9%) Results for 141 PNSP isolates tested with antimicrobial agents other than PEN (non susceptible): - CLI (2.1%), CHL (14.2%), ERY (6.4%), MDR (24.8%), SXT (82.3%), and TET (10.6%).
[39]	July 2000 to May 2001	Salvador	NP swab Colonization	33 children aged < 5 years; 43 children aged 5–17 years, and 19 adults aged > 17 years	Slum in Northeastern Amaralina.	95 isolates Non-susceptibility frequencies: - CHL (3%), MDR (5%), PEN (9%), ERY (2%), SXT (39%), and TET (15%).
[40]	March to June 2010	Niterói	NP swab Colonization	Children aged ≤ 6 years	Children at a daycare center (*n* = 102) and at the emergency room of a pediatric hospital (*n* = 140).	121 isolates Full susceptibility to CLI, LEV, RIF, and VAN. Non-susceptibility frequencies: - CHL (3.3%), ERY (1.7%), SXT (51.2%), and TET (8.3%). - PNSP: 27.3%, with MICs of 0.12–4 μg/mL.
[43]	9 April 2002 to 28 February 2003	São Paulo	NP swab Colonization	From 4 months to 17 years (average and standard deviation of 6.8 ± 4.7 years)	Children with sickle cell disease being followed up at Hospital São Paulo.	14 isolates Full susceptibility to CTX, ERY, and VAN. Non-susceptibility frequencies: - OXA disc screening: 35.7% non-susceptible to PEN; MIC = 0.25 µg/mL; LEV (42.9%), and SXT (64.3%).
[47]	Winters of 2000 and 2001 (May–August)	Goiânia	NP swab Colonization	Children aged < 5 years	20 large pediatric hospitals with healthy children from local childcare program.	227 isolates - PNSP: 19.8%.
CHL = chloramphenicol; CLI = clindamycin; CTX = ceftriaxone; ERY = erythromycin; IPD = invasive pneumococcal disease; LEV = levofloxacin; LIN = linezolid; MIC = minimum inhibitory concentration; MDR = multidrug-resistant (isolate resistant to three or more classes of antimicrobial agents); NIPD = non-invasive pneumococcal disease; NP = nasopharyngeal; OP = oropharyngeal; OXA = oxacillin; PCV10 = 10-valent pneumococcal conjugate vaccine; PEN = penicillin; PNSP = penicillin non-susceptible pneumococci; SXT = sulfamethoxazole-trimethoprim; TET = tetracycline; VAN = vancomycin.						
(b)						
[38]	29 September to 5 December 2014	Niterói	NP swab Colonization	Children aged ≥ 2 months and <6 years	Pediatric (2 private and 1 public) clinics.	9 isolates - Full susceptibility to CHL, LEV, RIF, and VAN. Non-susceptibility frequencies: - ERY (22.2%) and MDR (33.3%). - PNSP: 44.4% (PEN and CTX MICs ranged from 0.12 to 4.0 μg/mL and 0.023–0.5 μg/mL, respectively).
[21]	29 September to 5 December 2014	Niterói	NP swab Colonization	Children aged ≤ 6 years	Pediatric (2 private and 1 public) clinics.	118 isolates - Full susceptibility to LEV, RIF, and VAN; Non-susceptibility frequencies: - CHL (1.7%), CLI (20.3%), ERY (28%), MDR (22%), SXT (39.8%), and TET (29.7%). - PNSP: 38.9% (PEN and CTX MICs ranged from 0.12 to 8.0 μg/mL and 0.012–1.0 μg/mL, respectively).
[41]	January to December, 2011	Fortaleza	NP swab Colonization	Children aged 20 to 65 months	14 municipal kindergartens.	162 isolates Full susceptibility to amoxicillin and CTX; Non-susceptibility frequencies: CLI (10.5%), ERY (13.6%), PNSP (27.7%), and SXT (100%).
[42]	October to December, 2016	Niterói	NP swab Colonization	Adults aged 18 to 89 years	Patients assisted at a public health center that serves the population of an urban slum.	35 isolates Full susceptibility to CHL, LEV, and VAN. Non-susceptibility frequencies: - CLI (5.7%), ERY (5.7%), MDR (11.4%), RIF (2.9%), SXT (31.4%), and TET (20%). - PNSP: 22.9% (PEN MICs of 0.38–1.5 μg/mL)
[22]	2 September to 17 December 2019	Niterói	NP swab Colonization	Children aged ≤ 6 years	Pediatric (2 private and 2 public) clinics	75 isolates Full susceptibility to LIN, LEV, RIF, and VAN. Non-susceptibility frequencies: - CHL (1.7%), CLI (24%), ERY (25.3%), azithromycin (25.3%), MDR (22.7%), SXT (41.3%), and TET (25.3%) - PNSP: 37.3% (PEN and CTX MICs ranged from 0.12–4.0 μg/mL and 0.064–4.0 μg/mL, respectively.).
[44]	August 2017	São Paulo	NP swab Colonization	Children aged 12 to <24 months	Recruitment during an immunization campaign in 20 public health units in 5 different regions.	348 isolates - MIC to PEN ≥ 0.12 mg/L: 62%; - MIC to CTX ≥ 1.0 mg/L: 6.9%. - MIC_90_ to PEN and CTX: 1.0 mg/L and 0.5 mg/L, respectively; - MIC_50_ to PEN and CTX: 0.12 mg/L and 0.06 mg/L, respectively.
[45]	April to August 2017 (visit 1) and September to December 2017 (visit 2).	São Paulo	NP swab Colonization	Visit 1: mean age 81.5 years; range: 60–102 years). Visit 2: mean age 81.9 years	Outpatients treated at the Geriatrics Division of the *Hospital das Clínicas* of the Faculty of Medicine of the University of São Paulo.	32 isolates - PEN-resistant: 9.4%, two with MIC = 0.125 mg/L and one with MIC = 2 mg/L; - CTX-resistant (MIC = 1 mg/L): 3.1%.
[46]	19 June 2018 to 29 January 2019	Niterói and Rio de Janeiro	NP and OP swabs Colonization	Adults aged ≥ 18 years	Patients with systemic lupus erythematosus at 2 teaching hospitals.	11 isolates Full susceptibility to CHL, LEV, RIF, and VAN. Non-susceptibility frequencies: - CLI (18.2%), ERY (27.3%), MDR (27.3%), PEN (36.4%), TET (36.4%), and SXT (9.1%).
CHL = chloramphenicol; CLI = clindamycin; CTX = ceftriaxone; ERY = erythromycin; IPD = invasive pneumococcal disease; LEV = levofloxacin; LIN = linezolid; MIC = minimum inhibitory concentration; MDR = multidrug-resistant (isolate resistant to three or more classes of antimicrobial agents); NIPD = non-invasive pneumococcal disease; NP = nasopharyngeal; OP = oropharyngeal; OXA = oxacillin; PCV10 = 10-valent pneumococcal conjugate vaccine; PEN = penicillin; PNSP = penicillin non-susceptible pneumococci; SXT = sulfamethoxazole-trimethoprim; TET = tetracycline; VAN = vancomycin.						
(c)						
**Reference**	**Collection Date**	**City of Study**	**Clinical Source**	**Age Range**	**Number of Isolates and Main Findings**	
[33]	2000–2010	Angra dos Reis, Niterói, Porto Alegre, Ribeirão Preto, Rio de Janeiro, and São Paulo	NP and OP swabs	Pre-PCV10 period Colonization	225 results for antimicrobial agents.	
[33]	2011–2017	Campos dos Goytacazes, Niterói, and Rio de Janeiro	NP and OP swabs	Post-PCV10 period Colonization	229 results for antimicrobial agents.	
[33]	2000–2007	Niterói and Rio de Janeiro	Empyema aspirate, spinal aspirate, blood culture, CSF, pericardial fluid, peritoneal fluid, pleural fluid, peritoneal fluid, blood (catheter), blood/long-term central catheter, blood/PICC type, blood/CSF, blood/peritoneal fluid, pleural cavity secretion, thoracic cavity secretion, chest tube secretion, peritoneal secretion, meningeal specimen.	Pre-PCV10 period IPD	39 results for antimicrobial agents.	
[33]	2000–2009	Niterói, Porto Alegre, and Rio de Janeiro	Ear abscess, cervical abscess, buttock abscess, nasal/eye abscess, bronchial aspirate, corneal aspirate, sinus aspirate, pulmonary aspirate, tracheal aspirate, sputum, aqueous humor, vitreous humor, bronchoalveolar lavage, auricular secretion, bronchial secretion, tear duct secretion, conjunctival secretion, wound secretion, ocular secretion, skin secretion, pulmonary secretion, postauricular secretion, tracheal secretion, rectal swab, corneal ulcer, urine.	Pre-PCV10 period NIPD	82 results for antimicrobial agents.	
[33]	2011–2015	Niterói, Porto Alegre, and Rio de Janeiro	Ear abscess, cervical abscess, buttock abscess, nasal/eye abscess, bronchial aspirate, corneal aspirate, sinus aspirate, pulmonary aspirate, tracheal aspirate, sputum, aqueous humor, vitreous humor, bronchoalveolar lavage, secretion, auricular secretion, bronchial secretion, tear duct secretion, conjunctival secretion, wound secretion, ocular secretion, skin secretion, pulmonary secretion, postauricular secretion, tracheal secretion, rectal swab, corneal ulcer, urine.	Post-PCV10 Period NIPD	27 results for antimicrobial agents.	
CSF = cerebrospinal fluid; IPD = invasive pneumococcal disease; NIPD = non-invasive pneumococcal disease; NP = nasopharyngeal; OP = oropharyngeal; PCV10 = 10-valent pneumococcal conjugate vaccine; PICC = peripherally inserted central catheter.						

**Table 3:** Data related to antimicrobial resistance evaluated in the pre-PCV10 period divided into colonizing, non-invasive, and invasive isolates.

Antimicrobial Agents	Colonization Isolates		Non-Invasive Isolates		Invasive Isolates		N
	S (%)	NS (%)	S (%)	NS (%)	S (%)	NS (%)	
Chloramphenicol	703 (8.8%)	33 (0.4%)	43 (0.5%)	4 (0.05%)	7155 (89.5%)	58 (0.7%)	7996
Erythromycin	719 (8.9%)	36 (0.4%)	49 (0.6%)	2 (0.02%)	6795 (85%)	391 (4.9%)	7992
SXT	284 (3.5%)	465 (5.8%)	10 (0.1%)	39 (0.5%)	2907 (36.3%)	4311 (53.8%)	8016
Vancomycin	645 (92.1%)	-	40 (5.7%)	-	15 (2.1%)	-	700
Clindamycin	635 (91.1%)	4 (0.6%)	42 (6%)	1 (0.1%)	14 (2%)	1 (0.1%)	697
Tetracycline	643 (78.8%)	98 (12%)	31 (3.8%)	21 (2.6%)	17 (2.1%)	6 (0.7%)	816
Rifampicin	498 (88%)	2 (0.3%)	47 (8.3%)	-	19 (3.3%)	-	566
Levofloxacin	557 (88.1%)	7 (1.1%)	48 (7.6%)	-	20 (3.2%)	-	632
Ofloxacin	92 (100%)	-	-	-	-	-	92
Cefotaxime	111 (83.5%)	4 (3%)	8 (6%)	3 (2.3%)	6 (4.5%)	1 (0.7%)	133
Cefuroxime	123 (79.4%)	12 (7.7%)	3 (1.9%)	8 (5.2%)	6 (3.9%)	3 (1.9%)	155
Meropenem	87 (72.5%)	13 (10.8%)	3 (2.5%)	8 (6.7%)	6 (5%)	3 (2.5%)	120
Linezolid	169 (89.4%)	-	11 (5.8%)	-	9 (4.8%)	-	189
Telithromycin	180 (93.3%)	-	8 (4.1%)	-	5 (2.6%)	-	193
Trovafloxacin	2 (40%)	-	2 (40%)	-	1 (20%)	-	5
Quinupristin- dalfopristin	69 (77.5%)	-	11 (12.4%)	-	9 (10.1%)	-	89
Amoxicillin	111 (81.6%)	6 (4.4%)	8 (5.9%)	3 (2.2%)	6 (4.4%)	2 (1.5%)	136

N = number of isolates; NS = non-susceptible (intermediate + resistant); S = susceptible; SXT = sulfamethoxazole-trimethoprim.

**Table 4:** Data related to antimicrobial resistance evaluated in the post-PCV10 introduction period divided into colonizing, non-invasive, and invasive isolates.

Antimicrobial Agents	Colonization Isolates		Non-Invasive Isolates		Invasive Isolates		N
	S (%)	NS (%)	S (%)	NS (%)	S (%)	NS (%)	
Chloramphenicol	516 (6.3%)	9 (0.1%)	22 (0.3%)	-	7621 (92.5%)	73 (0.9%)	8241
Erythromycin	596 (6.9%)	131 (1.5%)	20 (0.2%)	3 (0.03%)	6397 (74.1%)	1487 (17.2%)	8634
SXT	362 (4.2%)	365 (4.2%)	15 (0.2%)	7 (0.08%)	4882 (56.8%)	2957 (34.4%)	8588
Vancomycin	525 (96%)	-	22 (4%)	-	-	-	547
Clindamycin	618 (87.9%)	63 (9%)	21 (3%)	1 (0.1%)	-	-	703
Tetracycline	436 (75.3%)	120 (20.7%)	16 (2.8%)	7 (1.2%)	-	-	579
Rifampicin	524 (95.8%)	1 (0.2%)	22 (4%)	-	-	-	547
Levofloxacin	565 (96.3%)	-	22 (3.7%)	-	-	-	587
Ofloxacin	-	-	-	-	-	-	-
Cefotaxime	73 (91.3%)	7 (8.7%)	-	-	-		80
Cefuroxime	18 (100%)	-	-	-	-	-	18
Meropenem	19 (51.4%)	5 (13.5%)	13 (35.1%)	-	-	-	37
Linezolid	99 (100%)	-	-	-	-	-	99
Telithromycin	82 (100%)	-	-	-	-	-	82
Trovafloxacin	-	-	-	-	-	-	-
Quinupristin- dalfopristin	24 (100%)	-	-	-	-	-	24
Amoxicillin	186 (97.4%)	5 (2.6%)	-	-	-	-	191

N = number of isolates; NS = non-susceptible (intermediate + resistant); S = susceptible; SXT = sulfamethoxazole-trimethoprim.

**Table 5:** (a) Data related to penicillin resistance in the pre-PCV10 period divided into meningitis, non-meningitis, and general parameters. (b) Data related to penicillin resistance in the post-PCV10 period divided into meningitis, non-meningitis, and general parameters.

**Antimicrobial Agent**	**Origin**	**Meningitis**			**Non Meningitis**			**General Parameter**		
		**S (%)**	**NS (%)**	**N**	**S (%)**	**NS (%)**	**N**	**S (%)**	**NS (%)**	**N**
(a)										
Penicillin	Colonization	-	-	-	-	-	-	1108 (74.1%)	387 (25.9%)	1495
	Non-invasive	-	-	-	-	-	-	51 (66.2%)	26 (33.8)	77
	Invasive	1527 (68.1%)	715 (31.9%)	2242	767 (82.3%)	165 (17.7%)	932	3082 (73.9%)	1087 (26.1%)	4169
(b)										
Penicillin	Colonization	-	-	-	-	-	-	539 (53.9%)	461 (46.1%)	1000
	Non-invasive	-	-	-	-	-	-	20 (83.3%)	4 (16.7%)	24
	Invasive	2545 (71.3%)	1025 (28.7%)	3570	4307 (93.7%)	291 (6.3%)	4598	-	-	-

N = number of isolates; NS = non-susceptible (intermediate + resistant); S = susceptible. The “general parameter” column refers to data prior to the change or not specified in academic productions.

**Table 6:** (a) Data related to ceftriaxone resistance in the pre-PCV10 period divided into meningitis, non-meningitis, and general parameters. (b) Data related to ceftriaxone resistance in the post-PCV10 introduction period divided into meningitis, non-meningitis, and general parameters.

Antimicrobial	Origin	Meningitis				Non Meningitis				General Parameter			
		S (%)	I (%)	R (%)	N	S (%)	I (%)	R (%)	N	S (%)	I (%)	R (%)	N
(a)													
Ceftriaxone	Colonization	-	-	-	-	-	5 (83.3%)	1 (16.7)	6	32 (100%)	-	-	32
	Non-invasive	-	-	-	-	5 (55.5%)	4 (44.4%)	-	9	-	-	-	-
	Invasive	1879 (87.7%)	174 (8.1%)	89 (4.1%)	2142	828 (92.8%)	62 (7%)	2 (0.2%)	892	3082 (96.9%)	87 (2.7%)	11 (0.3%)	3180
(b)													
Ceftriaxone	Colonization	-	-	-	-	98 (96.1%)	4 (3.9%)	-	102	517 (95.4%)	-	25 (4.6%)	542
	Non-invasive	-	-	-	-	-	-	-	-	-	-	-	-
	Invasive	3121 (89.7%)	239 (6.9%)	118 (3.4%)	3478	4244 (96.3%)	164 (3.7%)	-	4408	-	-	-	-

N = number of isolates; I = intermediate; R = resistant; S = susceptible. The “general parameter” column refers to data prior to the change in interpretation criteria for beta-lactams or not specified in academic productions.

**Table 7:** Criteria used for article selection.

Number	Exclusion Criteria	Inclusion Criteria
1	Review/Commentary Articles	Articles with raw data
2	Veterinary or plant isolates	Human isolates
3	Other bacterial species	*Streptococcus pneumoniae*
4	Out of date (before the year 2000)	Articles with data from 2000 to 2023
5	SIREVA II data	Data not presented by SIREVA II
6	Data from other countries	Brazilian data
7	Other unrelated topics	Articles within the proposed theme

SIREVA (Regional Surveillance System) is a compilation of data on *Haemophilus influenzae*, *Neisseria meningitidis,* and *Streptococcus pneumoniae* from Latin American countries since 2000.

## Supplementary Materials

The following supporting information can be downloaded at: https://www.mdpi.com/article/10.3390/antibiotics13010066/s1, File S1: Article search strategies according to the database; File S2: Articles included after manual search.
