# Supplementary material for: Antimicrobial Resistance in Streptococcus pneumoniae before and after the Introduction of Pneumococcal Conjugate Vaccines in Brazil: A Systematic Review
Source: Antibiotics (Basel). 2024 Jan 9;13(1):66. doi: 10.3390/antibiotics13010066 (PMC10812409; doi:10.3390/antibiotics13010066)
Supplement: Supplementary file 1 [file antibiotics-13-00066-s001.zip › antibiotics-2765166-SI.pdf]

Supplementary File 1. Article search strategies according to the database.

PUBMED

# 1	(((((((((((((((((((("Streptococcus pneumoniae"[MeSH Terms]) OR ("Streptococcus pneumoniae"[Title/Abstract])) OR ("Pneumococcal Infections"[MeSH Terms])) OR ("Pneumococcal Infections"[Title/Abstract])) OR ("Meningitis, Pneumococcal"[MeSH Terms])) OR ("Meningitis, Pneumococcal"[Title/Abstract])) OR ("Pneumonia, Pneumococcal"[MeSH Terms])) OR ("Pneumonia, Pneumococcal"[Title/Abstract])) OR (Pneumococcus[Title/Abstract])) OR ("Diplococcus pneumoniae"[Title/Abstract])) OR ("Streptococcus pneumoniae Infections"[Title/Abstract])) OR ("Streptococcus pneumoniae Infection"[Title/Abstract])) OR ("Pneumococcal Diseases"[Title/Abstract])) OR ("Pneumococcal Disease"[Title/Abstract])) OR ("Pneumococcal Infection"[Title/Abstract])) OR ("Pneumococcal Meningitides"[Title/Abstract])) OR ("Pneumococcal Meningitis"[Title/Abstract])) OR ("Pneumococcal Pneumonia"[Title/Abstract])) OR ("Pneumococcal Pneumonias"[Title/Abstract])) OR (Pneumococci[Title/Abstract])) OR ("S. pneumoniae"[Title/Abstract])
# 2	((((((((((((((((((((((((((((((((((((((("Drug resistance, bacterial"[MeSH Terms]) OR ("Drug resistance, bacterial"[Title/Abstract])) OR ("Drug resistance, multiple"[MeSH Terms])) OR ("Drug resistance, multiple"[Title/Abstract])) OR ("Drug resistance, multiple, bacterial"[MeSH Terms])) OR ("Drug resistance, multiple, bacterial"[Title/Abstract])) OR ("Tetracycline Resistance"[MeSH Terms])) OR ("Tetracycline Resistance"[Title/Abstract])) OR ("Penicillin Resistance"[MeSH Terms])) OR ("Penicillin Resistance"[Title/Abstract])) OR ("Vancomycin Resistance"[MeSH Terms])) OR ("Vancomycin Resistance"[Title/Abstract])) OR ("β-lactam resistance"[MeSH Terms])) OR ("β-lactam resistance"[Title/Abstract])) OR ("Cephalosporin Resistance"[MeSH Terms])) OR ("Cephalosporin Resistance"[Title/Abstract])) OR ("Chloramphenicol Resistance"[MeSH Terms])) OR ("Chloramphenicol Resistance"[Title/Abstract])) OR ("Antibacterial Drug Resistance"[Title/Abstract])) OR ("Multiple Drug Resistance"[Title/Abstract])) OR ("S. pneumoniae susceptibility"[Title/Abstract])) OR ("Multidrug Resistance"[Title/Abstract])) OR ("Multi-Drug Resistance"[Title/Abstract])) OR ("Penicillin Resistances"[Title/Abstract])) OR ("beta Lactam Resistance"[Title/Abstract])) OR ("beta-Lactamase Resistant"[Title/Abstract])) OR ("beta Lactamase Resistant"[Title/Abstract])) OR ("beta-Lactam Resistant"[Title/Abstract])) OR ("beta Lactam Resistant"[Title/Abstract])) OR ("beta-Lactamase Resistance"[Title/Abstract])) OR ("beta Lactamase Resistance"[Title/Abstract])) OR ("Antibacterial Drug Resistance"[Title/Abstract])) OR ("Antibiotic Resistance, Bacterial"[Title/Abstract])) OR ("Cephalosporin Resistances"[Title/Abstract])) OR ("Multiple Drug Resistance"[Title/Abstract])) OR ("Multidrug Resistance"[Title/Abstract])) OR ("Multi-Drug Resistance"[Title/Abstract])) OR ("Multiple Antibacterial Drug Resistance"[Title/Abstract])) OR ("Antibacterial Drug Resistance"[Title/Abstract])) OR ("Chloramphenicol Resistances"[Title/Abstract])
# 3	((((((((((((Amoxicillin[MeSH Terms]) OR (Amoxicillin[Title/Abstract])) OR ("Ceftaroline fosamil"[MeSH Terms])) OR ("Ceftaroline fosamil"[Title/Abstract])) OR (Cephalosporins[MeSH Terms])) OR (Cephalosporins[Title/Abstract])) OR (Doripenem[MeSH Terms])) OR (Doripenem[Title/Abstract])) OR (Carbapenems[MeSH Terms])) OR (Carbapenems[Title/Abstract])) OR ("beta-Lactams"[MeSH Terms])) OR

	("beta-Lactams"[Title/Abstract])) OR (Doxycycline[MeSH Terms])) OR (Doxycycline[Title/Abstract])) OR (Lefamulin[MeSH Terms])) OR (Lefamulin[Title/Abstract])) OR (Tetracycline[MeSH Terms])) OR (Tetracycline[Title/Abstract])) OR (Tetracyclines[MeSH Terms])) OR (Tetracyclines[Title/Abstract])) OR (Erythromycin[MeSH Terms])) OR (Erythromycin[Title/Abstract])) OR (Macrolides[MeSH Terms])) OR (Macrolides[Title/Abstract])) OR (Penicillins[MeSH Terms])) OR (Penicillins[Title/Abstract])) OR ("Trimethoprim, Sulfamethoxazole Drug Combination"[MeSH Terms])) OR ("Trimethoprim, Sulfamethoxazole Drug Combination"[Title/Abstract])) OR (Cefepime[MeSH Terms])) OR (Cefepime[Title/Abstract])) OR (Cefotaxime[MeSH Terms])) OR (Cefotaxime[Title/Abstract])) OR (Ceftriaxone[MeSH Terms])) OR (Ceftriaxone[Title/Abstract])) OR (Clindamycin[MeSH Terms])) OR (Clindamycin[Title/Abstract])) OR (Levofloxacin[MeSH Terms])) OR (Levofloxacin[Title/Abstract])) OR (Moxifloxacin[MeSH Terms])) OR (Moxifloxacin[Title/Abstract])) OR (Meropenem[MeSH Terms])) OR (Meropenem[Title/Abstract])) OR (Vancomycin[MeSH Terms])) OR (Vancomycin[Title/Abstract])) OR (Glycopeptides[MeSH Terms])) OR (Glycopeptides[Title/Abstract])) OR (Cefuroxime[MeSH Terms])) OR (Cefuroxime[Title/Abstract])) OR (Chloramphenicol[MeSH Terms])) OR (Chloramphenicol[Title/Abstract])) OR (Ertapenem[MeSH Terms])) OR (Ertapenem[Title/Abstract])) OR (Imipenem[MeSH Terms])) OR (Imipenem[Title/Abstract])) OR (Linezolid[MeSH Terms])) OR (Linezolid[Title/Abstract])) OR (Rifampin[MeSH Terms])) OR (Rifampin[Title/Abstract])) OR (Cefaclor[MeSH Terms])) OR (Cefaclor[Title/Abstract])) OR (Cefdinir[MeSH Terms])) OR (Cefdinir[Title/Abstract])) OR (Cefpodoxime[MeSH Terms])) OR (Cefpodoxime[Title/Abstract])) OR (Cefprozil[MeSH Terms])) OR (Cefprozil[Title/Abstract])) OR (Loracarbef[MeSH Terms])) OR (Loracarbef[Title/Abstract])) OR (Azithromycin[MeSH Terms])) OR (Azithromycin[Title/Abstract])) OR (Clarithromycin[MeSH Terms])) OR (Clarithromycin[Title/Abstract])) OR (Dirithromycin[MeSH Terms])) OR (Dirithromycin[Title/Abstract])) OR (Gemifloxacin[MeSH Terms])) OR (Gemifloxacin[Title/Abstract])) OR (Gatifloxacin[MeSH Terms])) OR (Gatifloxacin[Title/Abstract])) OR (Sparfloxacin[MeSH Terms])) OR (Sparfloxacin[Title/Abstract])) OR ("Quinupristin-dalfopristin"[MeSH Terms])) OR ("Quinupristin-dalfopristin"[Title/Abstract])) OR (Ofloxacin[MeSH Terms])) OR (Ofloxacin[Title/Abstract])) OR (Ciprofloxacin[MeSH Terms])) OR (Ciprofloxacin[Title/Abstract])) OR (Telithromycin[MeSH Terms])) OR (Telithromycin[Title/Abstract])) OR (Amoxycillin[Title/Abstract])) OR (Amoxicilline[Title/Abstract])) OR ("beta Lactams"[Title/Abstract])) OR ("beta-Lactam"[Title/Abstract])) OR ("beta Lactam"[Title/Abstract])) OR (Penicillin[Title/Abstract])) OR ("Trimethoprim-Sulfamethoxazole"[Title/Abstract])) OR ("Trimethoprim Sulfamethoxazole"[Title/Abstract])) OR ("Sulfamethoxazole-Trimethoprim Combination"[Title/Abstract])) OR ("Sulfamethoxazole Trimethoprim Combination"[Title/Abstract])) OR ("Trimethoprim-Sulfamethoxazole Combination"[Title/Abstract])) OR ("Trimethoprim Sulfamethoxazole Combination"[Title/Abstract])) OR (Cefepim[Title/Abstract])) OR (Cephotaxim[Title/Abstract])) OR (Cefotaxim[Title/Abstract])) OR (Ceftriaxon[Title/Abstract])) OR (Cefatriaxone[Title/Abstract])) OR (Dalacin C[Title/Abstract])) OR (Chlolincocin[Title/Abstract])) OR

	(Glycopeptide[Title/Abstract])) OR (Cephuroxime[Title/Abstract])) OR (Kloramfenikol[Title/Abstract])) OR (Cloranfenicol[Title/Abstract])) OR (Chlornitromycin[Title/Abstract])) OR (Linezolide[Title/Abstract])) OR (Rifampicin[Title/Abstract])) OR (Azythromycin[Title/Abstract])) OR (Gatifloxacine[Title/Abstract])) OR (Ofloxacine[Title/Abstract])
#4	((Brazil[MeSH Terms]) OR (Brazil[Title/Abstract])) OR (Brazilian[Title/Abstract])

Research strategy: ((#1) AND (#2)) AND (#3) AND (#4))

EMBASE

#1	'streptococcus pneumoniae'/exp OR 'streptococcus pneumoniae':ti,ab,kw OR 'pneumococcal infection'/exp OR 'pneumococcal infection':ti,ab,kw OR 'pneumococcal meningitis'/exp OR 'pneumococcal meningitis':ti,ab,kw OR 'pneumococci'/exp OR pneumococci:ti,ab,kw OR 'pneumococcal infections':ti,ab,kw OR 'meningitis, pneumococcal':ti,ab,kw OR 'pneumonia, pneumococcal':ti,ab,kw OR 'pneumococcus':ti,ab,kw OR 'diplococcus pneumoniae':ti,ab,kw OR 'streptococcus pneumoniae infections':ti,ab,kw OR 'streptococcus pneumoniae infection':ti,ab,kw OR 'pneumococcal diseases':ti,ab,kw OR 'pneumococcal disease':ti,ab,kw OR 'pneumococcal meningitides':ti,ab,kw OR 'pneumococcal pneumonia':ti,ab,kw OR 'pneumococcal pneumonias':ti,ab,kw OR 's. pneumoniae':ti,ab,kw
#2	'multidrug resistance'/exp OR 'multidrug resistance':ti,ab,kw OR 'tetracycline resistance'/exp OR 'tetracycline resistance':ti,ab,kw OR 'penicillin resistance'/exp OR 'penicillin resistance':ti,ab,kw OR 'vancomycin resistance'/exp OR 'vancomycin resistance':ti,ab,kw OR 'beta-lactam resistance'/exp OR 'beta-lactam resistance':ti,ab,kw OR 'cephalosporin resistance'/exp OR 'cephalosporin resistance':ti,ab,kw OR 'chloramphenicol resistance'/exp OR 'chloramphenicol resistance':ti,ab,kw OR 'drug resistance, bacterial':ti,ab,kw OR 'drug resistance, multiple':ti,ab,kw OR 's. pneumoniae susceptibility':ti,ab,kw OR 'drug resistance, multiple, bacterial':ti,ab,kw OR 'penicillin resistances':ti,ab,kw OR 'β-lactam resistance':ti,ab,kw OR 'beta lactam resistance':ti,ab,kw OR 'beta-lactamase resistant':ti,ab,kw OR 'beta lactamase resistant':ti,ab,kw OR 'beta-lactam resistant':ti,ab,kw OR 'beta lactam resistant':ti,ab,kw OR 'beta-lactamase resistance':ti,ab,kw OR 'beta lactamase resistance':ti,ab,kw OR 'antibiotic resistance, bacterial':ti,ab,kw OR 'cephalosporin resistances':ti,ab,kw OR 'multiple drug resistance':ti,ab,kw OR 'multi-drug resistance':ti,ab,kw OR 'multiple antibacterial drug resistance':ti,ab,kw OR 'antibacterial drug resistance':ti,ab,kw OR 'chloramphenicol resistances':ti,ab,kw
#3	'amoxicillin'/exp OR amoxicillin:ti,ab,kw OR 'ceftaroline fosamil'/exp OR 'ceftaroline fosamil':ti,ab,kw OR 'doripenem'/exp OR 'doripenem':ti,ab,kw OR 'beta lactam'/exp OR 'beta lactam':ti,ab,kw OR 'doxycycline'/exp OR 'doxycycline':ti,ab,kw OR 'lefamulin'/exp OR 'lefamulin':ti,ab,kw OR 'tetracycline'/exp OR 'tetracycline':ti,ab,kw OR 'erythromycin'/exp OR 'erythromycin':ti,ab,kw OR 'cefepime'/exp OR 'cefepime':ti,ab,kw OR 'cefotaxime'/exp OR 'cefotaxime':ti,ab,kw OR 'ceftriaxone'/exp OR 'ceftriaxone':ti,ab,kw OR 'clindamycin'/exp OR 'clindamycin':ti,ab,kw OR

	'levofloxacin'/exp OR 'levofloxacin':ti,ab,kw OR 'moxifloxacin'/exp OR 'moxifloxacin':ti,ab,kw OR 'meropenem'/exp OR 'meropenem':ti,ab,kw OR 'vancomycin'/exp OR 'vancomycin':ti,ab,kw OR 'glycopeptide'/exp OR 'glycopeptide':ti,ab,kw OR 'cefuroxime'/exp OR 'cefuroxime':ti,ab,kw OR 'chloramphenicol'/exp OR 'chloramphenicol':ti,ab,kw OR 'ertapenem'/exp OR 'ertapenem':ti,ab,kw OR 'imipenem'/exp OR 'imipenem':ti,ab,kw OR 'linezolid'/exp OR 'linezolid':ti,ab,kw OR 'rifampicin'/exp OR rifampicin:ti,ab,kw OR 'cefaclor'/exp OR 'cefaclor':ti,ab,kw OR 'cefdinir'/exp OR 'cefdinir':ti,ab,kw OR 'cefpodoxime'/exp OR 'cefpodoxime':ti,ab,kw OR 'cefprozil'/exp OR 'cefprozil':ti,ab,kw OR 'loracarbef'/exp OR 'loracarbef':ti,ab,kw OR 'azithromycin'/exp OR 'azithromycin':ti,ab,kw OR 'clarithromycin'/exp OR 'clarithromycin':ti,ab,kw OR 'dirithromycin'/exp OR 'dirithromycin':ti,ab,kw OR 'gemifloxacin'/exp OR 'gemifloxacin':ti,ab,kw OR 'gatifloxacin'/exp OR 'gatifloxacin':ti,ab,kw OR 'sparfloxacin'/exp OR 'sparfloxacin':ti,ab,kw OR 'ofloxacin'/exp OR 'ofloxacin':ti,ab,kw OR 'ciprofloxacin'/exp OR 'ciprofloxacin':ti,ab,kw OR 'telithromycin'/exp OR 'telithromycin':ti,ab,kw OR 'amoxycillin':ti,ab,kw OR 'amoxicilline':ti,ab,kw OR 'cephalosporins':ti,ab,kw OR 'carbapenems':ti,ab,kw OR 'beta-lactams':ti,ab,kw OR 'beta lactams':ti,ab,kw OR 'beta-lactam':ti,ab,kw OR 'tetracyclines':ti,ab,kw OR 'macrolides':ti,ab,kw OR 'penicillins':ti,ab,kw OR 'penicillin':ti,ab,kw OR 'trimethoprim, sulfamethoxazole drug combination':ti,ab,kw OR 'trimethoprim-sulfamethoxazole':ti,ab,kw OR 'trimethoprim sulfamethoxazole':ti,ab,kw OR 'sulfamethoxazole-trimethoprim combination':ti,ab,kw OR 'sulfamethoxazole trimethoprim combination':ti,ab,kw OR 'trimethoprim-sulfamethoxazole combination':ti,ab,kw OR 'trimethoprim sulfamethoxazole combination':ti,ab,kw OR 'cefepim':ti,ab,kw OR 'cephotaxim':ti,ab,kw OR 'cefotaxim':ti,ab,kw OR 'ceftriaxon':ti,ab,kw OR 'cefatriaxone':ti,ab,kw OR 'dalacin c':ti,ab,kw OR 'chlolincocin':ti,ab,kw OR 'glycopeptides':ti,ab,kw OR 'cephuroxime':ti,ab,kw OR 'kloramfenikol':ti,ab,kw OR 'cloranfenicol':ti,ab,kw OR 'chlornitromycin':ti,ab,kw OR 'linezolide':ti,ab,kw OR 'rifampin':ti,ab,kw OR 'azythromycin':ti,ab,kw OR 'gatifloxacine':ti,ab,kw OR 'quinupristin-dalfopristin':ti,ab,kw OR 'ofloxacine':ti,ab,kw
#4	'brazil'/exp OR 'brazil':ti,ab,kw OR 'brazilian'/exp OR 'brazilian':ti,ab,kw

Research strategy: ((#1) AND (#2)) AND (#3) AND (#4))

<u>SCOPUS</u>

#1	(TITLE-ABS-KEY ("Streptococcus pneumoniae") OR TITLE-ABS-KEY ("Pneumococcal Infections") OR TITLE-ABS-KEY ("Meningitis, Pneumococcal") OR TITLE-ABS-KEY ("Pneumonia, Pneumococcal") OR TITLE-ABS-KEY (pneumococcus) OR TITLE-ABS-KEY ("Diplococcus pneumoniae") OR TITLE-ABS-KEY ("Streptococcus pneumoniae Infections") OR TITLE-ABS-KEY ("Streptococcus pneumoniae Infection") OR TITLE-ABS-KEY ("Pneumococcal Diseases") OR TITLE-ABS-KEY ("Pneumococcal Disease") OR TITLE-ABS-KEY ("Pneumococcal Infection") OR TITLE-ABS-KEY ("Pneumococcal Meningitides") OR TITLE-ABS-KEY ("Pneumococcal Meningitis") OR TITLE-ABS-KEY ("Pneumococcal

	Pneumonia") OR TITLE-ABS-KEY ("Pneumococcal Pneumonias") OR TITLE-ABS-KEY (pneumococci) OR TITLE-ABS-KEY ("S. pneumoniae"))
#2	(TITLE-ABS-KEY ("Drug resistance, bacterial") OR TITLE-ABS-KEY ("Antibacterial Drug Resistance") OR TITLE-ABS-KEY ("Drug resistance, multiple") OR TITLE-ABS-KEY ("Multiple Drug Resistance") OR TITLE-ABS-KEY ("S. pneumoniae susceptibility") OR TITLE-ABS-KEY ("Multidrug Resistance") OR TITLE-ABS-KEY ("Multi-Drug Resistance") OR TITLE-ABS-KEY ("Drug resistance, multiple, bacterial") OR TITLE-ABS-KEY ("Tetracycline Resistance") OR TITLE-ABS-KEY ("Penicillin Resistance") OR TITLE-ABS-KEY ("Penicillin Resistances") OR TITLE-ABS-KEY ("Vancomycin Resistance") OR TITLE-ABS-KEY ("β-lactam resistance") OR TITLE-ABS-KEY ("beta Lactam Resistance") OR TITLE-ABS-KEY ("beta-Lactamase Resistant") OR TITLE-ABS-KEY ("beta Lactamase Resistant") OR TITLE-ABS-KEY ("beta-Lactam Resistant") OR TITLE-ABS-KEY ("beta Lactam Resistant") OR TITLE-ABS-KEY ("beta-Lactamase Resistance") OR TITLE-ABS-KEY ("beta Lactamase Resistance") OR TITLE-ABS-KEY ("Antibacterial Drug Resistance") OR TITLE-ABS-KEY ("Antibiotic Resistance, Bacterial") OR TITLE-ABS-KEY ("Cephalosporin Resistance") OR TITLE-ABS-KEY ("Cephalosporin Resistances") OR TITLE-ABS-KEY ("Multiple Drug Resistance") OR TITLE-ABS-KEY ("Multidrug Resistance") OR TITLE-ABS-KEY ("Multi-Drug Resistance") OR TITLE-ABS-KEY ("Multiple Antibacterial Drug Resistance") OR TITLE-ABS-KEY ("Antibacterial Drug Resistance") OR TITLE-ABS-KEY ("Chloramphenicol Resistance") OR TITLE-ABS-KEY ("Chloramphenicol Resistances"))
#3	(TITLE-ABS-KEY (amoxicillin) OR TITLE-ABS-KEY (amoxycillin) OR TITLE-ABS-KEY (amoxicilline) OR TITLE-ABS-KEY ("Ceftaroline fosamil") OR TITLE-ABS-KEY (cephalosporins) OR TITLE-ABS-KEY (doripenem) OR TITLE-ABS-KEY (carbapenems) OR TITLE-ABS-KEY ("beta Lactams") OR TITLE-ABS-KEY ("beta-Lactam") OR TITLE-ABS-KEY ("beta Lactam") OR TITLE-ABS-KEY (doxycycline) OR TITLE-ABS-KEY (lefamulin) OR TITLE-ABS-KEY (tetracycline) OR TITLE-ABS-KEY (tetracyclines) OR TITLE-ABS-KEY (erythromycin) OR TITLE-ABS-KEY (macrolides) OR TITLE-ABS-KEY (penicillins) OR TITLE-ABS-KEY (penicillin) OR TITLE-ABS-KEY ("Trimethoprim, Sulfamethoxazole Drug Combination") OR TITLE-ABS-KEY ("Trimethoprim-Sulfamethoxazole") OR TITLE-ABS-KEY ("Trimethoprim Sulfamethoxazole") OR TITLE-ABS-KEY ("Sulfamethoxazole-Trimethoprim Combination") OR TITLE-ABS-KEY ("Sulfamethoxazole Trimethoprim Combination") OR TITLE-ABS-KEY ("Trimethoprim-Sulfamethoxazole Combination") OR TITLE-ABS-KEY ("Trimethoprim Sulfamethoxazole Combination") OR TITLE-ABS-KEY (cefepime) OR TITLE-ABS-KEY (cefepim) OR TITLE-ABS-KEY (cefotaxime) OR TITLE-ABS-KEY (cephotaxim) OR TITLE-ABS-KEY (cefotaxim) OR TITLE-ABS-KEY (ceftriaxone) OR TITLE-ABS-KEY (ceftriaxon) OR TITLE-ABS-KEY (cefatriaxone) OR TITLE-ABS-KEY (clindamycin) OR TITLE-ABS-KEY (dalacin AND c) OR TITLE-ABS-KEY (chlolincocin) OR TITLE-ABS-KEY (levofloxacin) OR TITLE-ABS-KEY (moxifloxacin) OR TITLE-ABS-KEY (meropenem) OR TITLE-ABS-

	KEY (vancomycin) OR TITLE-ABS-KEY (glycopeptides) OR TITLE-ABS-KEY (glycopeptide) OR TITLE-ABS-KEY (cefuroxime) OR TITLE-ABS-KEY (cephuroxime) OR TITLE-ABS-KEY (chloramphenicol) OR TITLE-ABS-KEY (kloramfenikol) OR TITLE-ABS-KEY (cloranfenicol) OR TITLE-ABS-KEY (chlornitromycin) OR TITLE-ABS-KEY (ertapenem) OR TITLE-ABS-KEY (imipenem) OR TITLE-ABS-KEY (linezolid) OR TITLE-ABS-KEY (linezolide) OR TITLE-ABS-KEY (rifampin) OR TITLE-ABS-KEY (rifampicin) OR TITLE-ABS-KEY (cefaclor) OR TITLE-ABS-KEY (cefdinir) OR TITLE-ABS-KEY (cefpodoxime) OR TITLE-ABS-KEY (cefprozil) OR TITLE-ABS-KEY (loracarbef) OR TITLE-ABS-KEY (azithromycin) OR TITLE-ABS-KEY (azythromycin) OR TITLE-ABS-KEY (clarithromycin) OR TITLE-ABS-KEY (dirithromycin) OR TITLE-ABS-KEY (gemifloxacin) OR TITLE-ABS-KEY (gatifloxacin) OR TITLE-ABS-KEY (gatifloxacine) OR TITLE-ABS-KEY (sparfloxacin) OR TITLE-ABS-KEY ("Quinupristin-dalfopristin") OR TITLE-ABS-KEY (ofloxacin) OR TITLE-ABS-KEY (ofloxacine) OR TITLE-ABS-KEY (ciprofloxacin) OR TITLE-ABS-KEY (telithromycin))
#4	TITLE-ABS-KEY (brazil) OR TITLE-ABS-KEY (brazilian))

Research strategy: ((#1) AND (#2)) AND (#3) AND (#4))

WEB OF SCIENCE

#1	(((((((((((((((TS=(“Streptococcus pneumoniae”)) OR TS=(“Pneumococcal Infections”)) OR TS=("Meningitis, Pneumococcal")) OR TS=("Pneumonia, Pneumococcal")) OR TS=(Pneumococcus)) OR TS=(“Diplococcus pneumoniae”)) OR TS=("Streptococcus pneumoniae Infections")) OR TS=("Streptococcus pneumoniae Infection")) OR TS=("Pneumococcal Diseases")) OR TS=("Pneumococcal Disease")) OR TS=("Pneumococcal Infection")) OR TS=(“Pneumococcal Meningitides”)) OR TS=(“Pneumococcal Meningitis”)) OR TS=(“Pneumococcal Pneumonia”)) OR TS=(“Pneumococcal Pneumonias”)) OR TS=(Pneumococci)) OR TS=("S. pneumoniae")
#2	((((((((((((((((((((((((((((((TS=("Drug resistance, bacterial")) OR TS=("Antibacterial Drug Resistance")) OR TS=("Drug resistance, multiple")) OR TS=("Multiple Drug Resistance")) OR TS=(“S. pneumoniae susceptibility”)) OR TS=("Multidrug Resistance")) OR TS=(“Multi-Drug Resistance”)) OR TS=(“Drug resistance, multiple, bacterial”)) OR TS=("Tetracycline Resistance")) OR TS=("Penicillin Resistance")) OR TS=("Penicillin Resistances")) OR TS=("Vancomycin Resistance")) OR TS=("β-lactam resistance")) OR TS=("beta Lactam Resistance")) OR TS=("beta-Lactamase Resistant")) OR TS=("beta Lactamase Resistant")) OR TS=("beta-Lactam Resistant")) OR TS=("beta Lactam Resistant")) OR TS=("beta-Lactamase Resistance")) OR TS=("beta Lactamase Resistance")) OR TS=("Antibacterial Drug Resistance")) OR TS=("Antibiotic Resistance, Bacterial")) OR TS=(“Cephalosporin Resistance”)) OR TS=("Cephalosporin Resistances")) OR TS=("Multiple Drug Resistance")) OR TS=("Multidrug Resistance")) OR TS=("Multi-Drug Resistance")) OR TS=("Multiple

	Antibacterial Drug Resistance")) OR TS=("Antibacterial Drug Resistance")) OR TS=("Chloramphenicol Resistance")) OR TS=("Chloramphenicol Resistances")
#3	((TS=(Amoxicillin)) OR TS=(Amoxycillin)) OR TS=(Amoxicilline)) OR TS=(“Ceftaroline fosamil”)) OR TS=(Cephalosporins)) OR TS=(Doripenem)) OR TS=(Carbapenems)) OR TS=("beta-Lactams")) OR TS=("beta Lactams")) OR TS=("beta-Lactam")) OR TS=(“beta Lactam”)) OR TS=(Doxycycline)) OR TS=(Lefamulin)) OR TS=(Tetracycline)) OR TS=(Tetracyclines)) OR TS=(Erythromycin)) OR TS=(Macrolides)) OR TS=(Penicillins)) OR TS=(Penicillin)) OR TS=("Trimethoprim, Sulfamethoxazole Drug Combination")) OR TS=("Trimethoprim-Sulfamethoxazole")) OR TS=("Trimethoprim Sulfamethoxazole")) OR TS=("Sulfamethoxazole-Trimethoprim Combination")) OR TS=("Sulfamethoxazole Trimethoprim Combination")) OR TS=(“Trimethoprim-Sulfamethoxazole Combination”)) OR TS=("Trimethoprim Sulfamethoxazole Combination")) OR TS=(Cefepime)) OR TS=(Cefepim)) OR TS=(Cefotaxime)) OR TS=(Cephotaxim)) OR TS=(Cefotaxim)) OR TS=(Ceftriaxone)) OR TS=(Ceftriaxon)) OR TS=(Cefatriaxone)) OR TS=(Clindamycin)) OR TS=(Dalacin C)) OR TS=(Chlolincocin)) OR TS=(Levofloxacin)) OR TS=(Moxifloxacin)) OR TS=(Meropenem)) OR TS=(Vancomycin)) OR TS=(Glycopeptides)) OR TS=(Glycopeptide)) OR TS=(Cefuroxime)) OR TS=(Cephuroxime)) OR TS=(Chloramphenicol)) OR TS=(Kloramfenikol)) OR TS=(Cloranfenicol)) OR TS=(Chlornitromycin)) OR TS=(Ertapenem)) OR TS=(Imipenem)) OR TS=(Linezolid)) OR TS=(Linezolide)) OR TS=(Rifampin)) OR TS=(Rifampicin)) OR TS=(Cefaclor)) OR TS=(Cefdinir)) OR TS=(Cefpodoxime)) OR TS=(Cefprozil)) OR TS=(Loracarbef)) OR TS=(Azithromycin)) OR TS=(Azythromycin)) OR TS=(Clarithromycin)) OR TS=(Dirithromycin)) OR TS=(Gemifloxacin)) OR TS=(Gatifloxacin)) OR TS=(Gatifloxacine)) OR TS=(Sparfloxacin)) OR TS=("Quinupristin-dalfopristin")) OR TS=(Ofloxacin)) OR TS=(Ofloxacine)) OR TS=(Ciprofloxacin)) OR TS=(Telithromycin)
#4	(TS=(Brazil)) OR TS=(Brazilian)

Research strategy: ((#1) AND (#2)) AND (#3) AND (#4))

LILACS

#1 AND #2 AND #3 AND #4	((Streptococcus pneumoniae) OR (Pneumococcal Infections) OR (Meningitis, Pneumococcal) OR (Pneumonia, Pneumococcal) OR (Pneumococcus) OR (Diplococcus pneumoniae) OR (Streptococcus pneumoniae Infection) OR (Streptococcus pneumoniae Infections) OR (Pneumococcal Disease) OR (Pneumococcal Diseases) OR (Pneumococcal Infection) OR (Pneumococcal Meningitides) OR (Pneumococcal Meningitis) OR (Pneumococcal Pneumonia) OR (Pneumococcal Pneumonias) OR (Pneumococci) OR ("S.

	pneumoniae")) AND ((Drug resistance, bacterial) OR (Antibacterial Drug Resistance) OR (Drug resistance, multiple) OR (Multiple Drug Resistance) OR (Multidrug Resistance) OR (Multi-Drug Resistance) OR (Drug resistance, multiple, bacterial) OR (Tetracycline Resistance) OR (Penicillin Resistance) OR (Vancomycin Resistance) OR (beta Lactam Resistance) OR (beta-Lactamase Resistant) OR (beta Lactamase Resistant) OR (beta-Lactam Resistant) OR (beta Lactam Resistant) OR (beta-Lactamase Resistance) OR (beta Lactamase Resistance) OR (Antibacterial Drug Resistance) OR (Antibiotic Resistance, Bacterial) OR (Cephalosporin Resistance) OR (Cephalosporin Resistances) OR (Multiple Drug Resistance) OR (Multidrug Resistance) OR (Multi-Drug Resistance) OR (Multiple Antibacterial Drug Resistance) OR (Antibacterial Drug Resistance) OR (Chloramphenicol Resistance) OR (Chloramphenicol Resistances) OR ("S. pneumoniae susceptibility") OR ("Penicillin Resistances") OR ("β-lactam resistance")) AND ((Amoxicillin) OR (Amoxycillin) OR (Amoxicilline) OR (Cephalosporins) OR (Doripenem) OR (Carbapenems) OR (beta-Lactams) OR (beta Lactams) OR (beta-Lactam) OR (beta Lactam) OR (Doxycycline) OR (Tetracycline) OR (Erythromycin) OR (Macrolides) OR (Penicillins) OR (Penicillin) OR (Trimethoprim, Sulfamethoxazole Drug Combination) OR (Trimethoprim-Sulfamethoxazole) OR (Trimethoprim Sulfamethoxazole) OR (Sulfamethoxazole-Trimethoprim Combination) OR (Sulfamethoxazole Trimethoprim Combination) OR (Trimethoprim-Sulfamethoxazole Combination) OR (Trimethoprim Sulfamethoxazole Combination) OR (Cefepime) OR (Cefepim) OR (Cefotaxime) OR (Cephotaxim) OR (Cefotaxim) OR (Ceftriaxone) OR (Ceftriaxon) OR (Cefatriaxone) OR (Clindamycin) OR (Dalacin C) OR (Chlolincocin) OR (Levofloxacin) OR (Moxifloxacin) OR (Meropenem) OR (Vancomycin) OR (Glycopeptides) OR (Glycopeptide) OR (Cefuroxime) OR (Cephuroxime) OR (Chloramphenicol) OR (Kloramfenikol) OR (Cloranfenicol) OR (Chlornitromycin) OR (Ertapenem) OR (Imipenem) OR (Linezolid) OR (Linezolide) OR (Rifampin) OR (Rifampicin) OR (Cefaclor) OR (Cefdinir) OR (Azithromycin) OR (Azythromycin) OR (Clarithromycin) OR (Gemifloxacin) OR (Gatifloxacin) OR (Gatifloxacine) OR (Ofloxacin) OR (Ofloxacine) OR (Ciprofloxacin) OR ("Ceftaroline fosamil") OR (Lefamulin) OR (Tetracyclines) OR (Cefpodoxime) OR (Cefprozil) OR (Loracarbef) OR (Dirithromycin) OR (Sparfloxacin) OR (Quinupristin-dalfopristin) OR (Telithromycin)) AND ((Brazil) OR (Brazilian))

Research strategy: ((#1) AND (#2)) AND (#3) AND (#4))

Supplementary File 2. Articles included after manual search.

1. FONSECA, P.B.B. et al. Colonização nasofaríngea pelo *Streptococcus pneumoniae* em crianças com doença falciforme usando penicilina profilática. Jornal de Pediatria (Rio J), v. 81, n. 2, p. 149-154, 2005.
Comments: Isolates from colonization of São Paulo from 2002 to 2003.

2. Brandileone MC de C, Zanella RC, Almeida SCG, Cassiolato AP, Lemos APS de, Salgado MM, et al. Long-term effect of 10-valent pneumococcal conjugate vaccine on nasopharyngeal carriage of Streptococcus pneumoniae in children in Brazil. Vaccine 2019;37:5357–63. https://doi.org/10.1016/j.vaccine.2019.07.043.
Comments: Isolates from colonization of children in São Paulo obtained during a vaccination campaign.

3. Zanella RC, de Cunto Brandileone MC, Almeida SCG, de Lemos APS, Sacchi CT, Gonçalves CR, et al. Nasopharyngeal carriage of *Streptococcus pneumoniae*, *Haemophilus influenzae*, and *Staphylococcus aureus* in a Brazilian elderly cohort. PLoS One 2019;14:1–13. https://doi.org/10.1371/journal.pone.0221525.
Comments: Isolates from colonization of elderly people in São Paulo.

4. Rezende RPV, Cardoso-Marques NT, Rodrigues LAS, Almeida JPCL, Pillegi GS, Teixeira LM, et al. Carriage prevalence, serotype distribution, and antimicrobial susceptibility among pneumococcal isolates recovered from adults with systemic lupus erythematosus. Lupus 2021;30:1863–5. https://doi.org/10.1177/09612033211030549.
Comments: Colonization isolates from lupus patients in Rio de Janeiro.
